# An Investigation into the Cognition Behind Spontaneous String Pulling in New Caledonian Crows

**DOI:** 10.1371/journal.pone.0009345

**Published:** 2010-02-22

**Authors:** Alex H. Taylor, Felipe S. Medina, Jennifer C. Holzhaider, Lindsay J. Hearne, Gavin R. Hunt, Russell D. Gray

**Affiliations:** Department of Psychology, University of Auckland, Auckland, New Zealand; Indiana University, United States of America

## Abstract

The ability of some bird species to pull up meat hung on a string is a famous example of spontaneous animal problem solving. The “insight” hypothesis claims that this complex behaviour is based on cognitive abilities such as mental scenario building and imagination. An operant conditioning account, in contrast, would claim that this spontaneity is due to each action in string pulling being reinforced by the meat moving closer and remaining closer to the bird on the perch. We presented experienced and naïve New Caledonian crows with a novel, visually restricted string-pulling problem that reduced the quality of visual feedback during string pulling. Experienced crows solved this problem with reduced efficiency and increased errors compared to their performance in standard string pulling. Naïve crows either failed or solved the problem by trial and error learning. However, when visual feedback was available via a mirror mounted next to the apparatus, two naïve crows were able to perform at the same level as the experienced group. Our results raise the possibility that spontaneous string pulling in New Caledonian crows may not be based on insight but on operant conditioning mediated by a perceptual-motor feedback cycle.

## Introduction

As early as the 16^th^ century it was noted that birds would pull up string to obtain food [1]. Studies with great tits (*Parus major*), European greenfinches (*Carduelis chloris*), canaries (*Serinus* spp.), chaffinches (*Fringilla coelebs*), budgerigars (*Melopsittacus undulates*), goldfinches (*Carduelis carduelis*), and siskins (*Carduelis spinus*), have suggested that such performances are based on trial and error learning [2]–[4]. A recent study with domestic dogs (*Canis lupis familiaris*) came to the same conclusion [5]. Similarly, Piaget [6] suggested that string pulling does not involve insightful actions.

However, corvids [7], [8] and psittacids [9], [10] have often succeeded at this famous example of animal problem solving within seconds of exposure to it. Complex cognitive mechanisms such as insight [7], [8] and imagination [11] have therefore been proposed to explain this spontaneous behaviour. Insight has been described as ‘mental scenario building’ where “…*alternative choices or motor patterns are expressed or suppressed depending on their probable outcome, either before or after such outcome has been experienced.*” [8]. Imagination is defined as the “…*simulation of scenarios not available to perception in the minds' eye.*” [11]. These mechanisms may require another form of ‘insight’ based on an understanding of the relation between the food and the string, or its ‘connectivity’ [6], [12], [13].

Recent experimental work on string pulling has focused on string discrimination tasks. Both ravens (*Corvus corax*) and keas (*Nestor notabilis*) are sensitive to the object at the end of the string and do not attempt to pull up items that are overly large in size [8], [10]. When faced with parallel slanted strings both ravens and keas pull the string connected to a reward rather than the unrewarded string directly above the food [8], [10]. However, when faced with crossed strings of the same colour only one raven was able to consistently choose the string connected to the meat rather than the string tied directly above the meat [8]. In contrast, five of the seven keas tested with differently-coloured crossed strings were able to choose the correct string [10]. However, when they were subsequently tested with same-coloured strings, only three keas continued to choose the correct string. The keas' performance with these two crossed-string tests suggests that they had used ‘path continuity’ as a visual cue for string selection [10] rather than an understanding of ‘connectivity’ [6], [12], [13].

Despite the claims that complex cognitive mechanisms such as insight are involved in spontaneous string pulling, only one experiment has attempted to manipulate an animal's performance with the standard string-pulling problem. Ravens with experience of string pulling were able to solve a counter-intuitive problem where a string had to be pulled down from a pivot rather than being pulled up from beneath a perch [14]. Ravens naïve to string pulling could not solve the problem. The authors suggested that the naïve ravens failed because of a lack of “…*counter-intuitive means-end understanding…*” [14]. However, this task required divided attention - ravens pulling and stepping on the string did not have the meat in their line of sight as in standard string pulling. In standard string pulling a positive perceptual-motor feedback cycle exists - pulling the string moves the meat towards an individual, and stepping on the string holds it in a position closer than before the pull. As the meat is always within sight, the effect of string pulling on the meat can be constantly monitored. Such feedback may drive spontaneous string-pulling performances if the sight of food moving and then staying closer to an individual after a series of actions acts as an internal psychological reinforcer and so increases motivation for the same actions to be repeated. For the naïve ravens this cycle may have been interrupted as they had to both look up to coordinate pulling the string down and look down to see the effects of their actions on the position of the meat. The need to split their attention may have prevented these ravens from being able to see that their actions had a positive effect on the meat's position. Experienced ravens may have solved this problem because they had already learnt to coordinate pull-step actions on the string. Therefore, they could focus their attention on the meat rather than on co-ordinating string pulling. Consequently, the experienced ravens solved the problem while the naïve ones did not.

**Figure 1:**
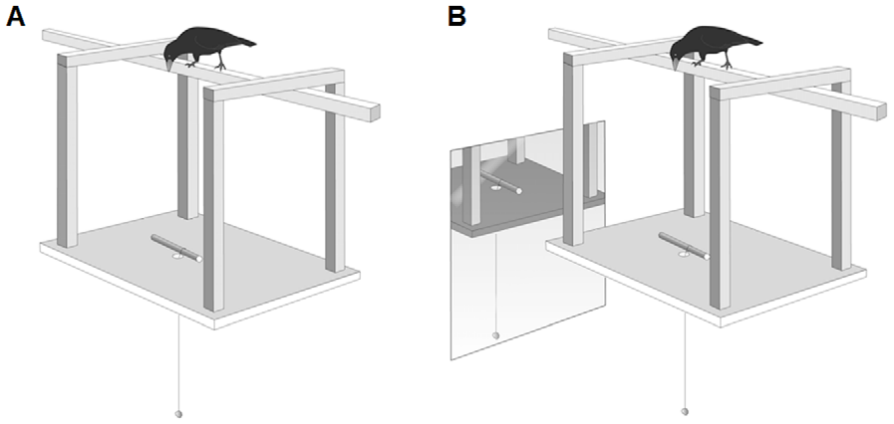
The visually-restricted apparatus. (A) Without the mirror. (B) With the mirror.

New Caledonian crows (*Corvus moneduloides*) exhibit exceptional tool skills both in the wild [15], [16] and in experimental situations [17]–[21], but they have not been tested on string-pulling tasks. We presented these crows with the standard string-pulling problem, a range of string discrimination problems [7], [8], [10] and a novel string-pulling task where the positive perceptual-motor feedback cycle was disrupted. To reduce the quality of visual feedback we suspended a string through a small hole in a horizontal sheet of plywood (Figure 1a). During string pulling a crow could only see the food on the end of the string from directly above the hole. If the crow moved away from the hole in the process of pulling up the string it lost visual feedback about the consequences of its string-pulling actions. The small diameter of the hole also changed the type of visual feedback received by the crows. In the standard string-pulling problem the crows could accurately judge whether a pull-step had moved the food closer by viewing the food from a side angle. With the novel apparatus the crows could only judge if the meat was moving closer from a head-on angle directly above the string and meat. This potentially prevented accurate estimation of whether the distance between crow and meat was reduced after a pull-step, and therefore a pull-step could appear to have a neutral effect on the meat's position rather than a positive one.

If New Caledonian crows spontaneously solved the standard string-pulling problem, we predicted that their performance on the visually-restricted task might show whether insight or a perceptual-motor feedback cycle was the cognitive mechanism behind such behaviour. If the crows were using insight, the reduced visual feedback the crows received when attempting to solve the visually-restricted problem should have little effect on their performance. This is because the crows would have already built a mental scenario in which they had imagined the effects of pulling and stepping on the string (i.e. it brought the meat closer) and so, during problem solving, could imagine the effects they were having on the string when perceptual feedback was not available or appeared neutral. The insight hypothesis therefore predicts little difference in the degree of the efficiency (calculated as the number of pulls followed by a step) or the rate of errors between standard and visually-restricted string pulling. It would also predict that crows naïve to string pulling could produce spontaneous solutions when presented with the visually-restricted apparatus, just as they do when faced with the standard string-pulling paradigm. Alternatively, if string pulling is mediated by perceptual-motor feedback experienced crows should be less efficient and make more errors when presented with the visually-restricted apparatus, and naïve crows should not produce spontaneous performances. The perceptual-motor feedback hypothesis also predicts that naïve crows would perform better in the visually-restricted condition if they had access to more information about the position of the meat during problem solving. To address this prediction, we provided naïve crows with access to a mirror (Figure 1b) so they could potentially follow the meat's movement from a side-on angle when pulling the string in the visual-restricted task.

## Materials and Methods

### Ethics Statement

Our work was carried out under University of Auckland Animal Ethics Committee approval R602.

### (a) Subjects

We carried out the experiments with 12 wild crows captured on the island of Maré, New Caledonia. We aged the crows using mouth colouration. Eleven of the crows were adults and one, Tiga, was a juvenile. The crows were housed in a 5-cage outdoor aviary close to the location of capture; the cages varied in size but were all at least 8 m^2^ in area and 3 m high.

After capture, a crow was left to get accustomed to the aviary and human presence for three days before experimental procedures began. Crows were habituated to string for 3 days before the experiment by tying string between perches in the cages. The experiments were only carried out with one crow at a time in a separate cage; the other crows could not see into the experimental cage.

### (b) Materials

#### (i) Materials used in the standard string-pulling and string discrimination problems

**Figure 2:**
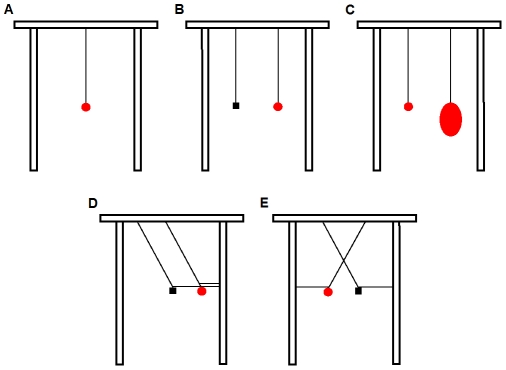
Standard string-pulling problem and string discrimination tasks. (A) Standard string-pulling problem. (B) Object discrimination test: circle  =  meat; rectangle  =  rock. (C) Overload test: small circle  =  meat; large circle  = 700 g chicken. (D) Slanted-string test. (E) Crossed-string test.

Standard string-pulling (meat suspended from a single, vertical string) and string discrimination tasks were conducted using a horizontal wooden perch 180 cm long and 5 cm in diameter. The perch was 2 m above the ground. In the standard string-pulling task, meat was attached to a 40 cm long length of string (2 mm in diameter) (Figure 2a). In object discrimination tests with parallel (Figure 2b,d) and crossed strings (Figure 2e), meat was attached to the end of one string and a small rock 2 cm in diameter was attached to the end of the other string. In the overload test, meat was attached to one string and a 700 g chicken carcass was attached to the second string (Figure 2c). Translucent fishing line was used to hold the strings in the slanted and crossed positions. We carried out three different crossed-string conditions using different combinations of string colour: (1) same-coloured strings (both strings were white), (2) different-coloured strings (one white, one blue), and (3) different-patterned strings (one had 1 mm black stripes marked every 3 mm on white string, and the other had 10 mm black stripes marked every 10 mm on white string). In all other tests only white string was used.

#### ii) Materials used in the visually-restricted string-pulling task

Visually-restricted string pulling was conducted on a plywood platform suspended under the perch (Figure 2a). The platform was 60 cm×60 cm and had a 3 cm diameter hole in its centre. Situated at the edge of the hole was a 15 cm length of branch 2 cm in diameter. Meat was suspended on a 40 cm length of string attached to the short section of branch. In the mirror condition, a mirror 50 cm square was attached to the wall of the aviary 70 cm from the platform (Figure 1b). It was positioned so that it was possible for a crow interacting with the string to see a reflection of itself, the string and the meat in the mirror.

### (c) General Procedure

Trials were given in blocks of 10, with no more than 2 blocks per day. All tests were recorded on video tape through the wall of an adjoining observation cage. The 12 crows were separated into three treatment groups with 4 birds in each group. The experienced and naïve groups contained four adults, while the mirror group contained three adults and a juvenile. The experienced group was given 10 standard string-pulling trials followed by 10 visually-restricted string-pulling trials. The naïve and mirror groups were given 10 visually-restricted string-pulling trials then 10 standard string-pulling trials. All the string discrimination tasks were given after the completion of the standard and visually-restricted string-pulling problems. Therefore, all crows were competent at string pulling before attempting the discrimination tests.

#### i) Standard string-pulling procedure

Crows were given 10 trials. A trial was scored from when the crow first touched the string until it left the perch or obtained the meat. We recorded the string-pulling technique and the number and type of errors. We also recorded the time taken from first contact with the string until the crow either obtained the food or lost interest.

#### (ii) Visually-restricted string-pulling procedure

We habituated crows to the visually-restricted apparatus by leaving it in their cages for three days just prior to the experiment. Crows were given 10 trials with the visually-restricted apparatus. A trial was scored from when a crow first touched the string to when it left the platform or obtained the meat. The experienced group was tested on the apparatus after completing 10 trials of standard string pulling. The naïve group was not given any standard string-pulling trials before being tested on the visually-restricted apparatus. The mirror group was first habituated to mirrors by having them placed in their cages for two weeks. This group was then given 20 familiarisation trials locating food with the use of a mirror (Medina *et al.* unpublished data). The crows in the mirror group were finally given 10 experimental trials with a mirror set up next to the visually-restricted apparatus (Figure 1b). If crows did not interact with the string within 10 mins they were retested the next day.

#### (iii) Procedure for string discrimination problems

Crows were given four string discrimination tasks. Birds received 20 trials on each problem. Two of the four problems involved vertical strings: (1) object discrimination (meat *vs.* rock; Figure 2b), and (2) overload (1 g meat *vs.* 700 g chicken; Figure 2c). In the other two problems crows had to choose one of two slanted (Figure 2d) or crossed (Figure 2e) strings: one string had meat on it and the other had a small rock. All 12 crows were given the object discrimination and slanted tests. For the crossed-string test the crows were split into three groups and allocated to the different conditions in the same groups used in the previous string-pulling tests: experienced, naïve and mirror. The experienced group was given the same-coloured condition, the naïve group the different-coloured condition and the mirror group the patterned condition. Only the naïve and mirror groups were given the overload test; the experienced group was not tested because the birds had to be released for the breeding season. In all four discrimination tests the position of the meat and the other object were alternated randomly between the two strings across trials. The string that each crow first interacted with was scored as its choice in each trial. The string discrimination tasks were presented in the following order: 1) object discrimination, 2) slanted-string, 3) crossed-string, and 4) overload.

### (d) Data Analysis

We followed the methodology of a previous study [10] when analysing a crow's first trial with standard string pulling, which allowed us to compare our results with those found in keas. That is, we looked at interaction behaviours with the string besides a pull followed by a step (pull-step). These other interactions consisted of single pulls, pecks and touching the string. First solution times were calculated using a cumulative score across trials. In our comparison with the kea study, we excluded data from the one juvenile kea that was tested due to its extended solution time over several sessions. Unfortunately, it was not possible to compare the crows' behaviour with those of other studies where complex cognition has been suggested because of methodological issues such as the use of juveniles [14], lack of controls for neophobia, competition and social learning [7] and small sample size [9].

To quantify variation in the efficiency of string-pulling, we devised a novel measure, the ‘pull-step ratio’. Both single pulls and pulls followed by a step were included in this measure. To successfully pull up the meat a crow needed to follow a pull with a step on the string to stop the string and meat falling back down. A high pull-step ratio indicates that a crow usually stepped on the string after pulling it up. A low pull-step ratio indicates that a crow performed many pulls and few steps. String pulling errors were defined as behaviours other than sequential pull-steps, pulls and pecks. These included (i) pulling the string then pushing it against the perch, (ii) stopping pull-step actions before the meat was obtained, (iii) taking the foot off the string after stepping, and (iv) attempting to stand on the string but mis-coordinating the step. All analyses of pull-step ratios and errors were counts across the entire trial, unless we indicate that they excluded the first pull-step. We excluded data before the first pull-step when comparing the experienced group's performance in standard and visually-restricted string pulling. We did this to remove variation due to exploratory behaviour and to allow a meaningful comparison of performances between standard and visually-restricted string pulling. Results are expressed as means ± s.e.m.

## Results

### (a) Standard String Pulling

The four crows in the experienced group showed immediate interest in the baited string and had no obvious neophobic response to the apparatus. Three of these four crows obtained the meat on their first trial (See Movie S1). Goo pecked and pulled at the string on its first trial, then solved the problem on the second trial. Two crows showed no string-interaction behaviours other than pull-steps before the first solution. Owl performed one behaviour and Goo performed 11 behaviours (grand mean ± s.e.m.: 3.25±2.63). First solution times ranged from 6–37 s (16.25±14.24) (trials 1 and 2 were combined for Goo's score). The performance of the four crows compared favourably with that of six adult keas [10]. Interaction behaviours for the keas (excluding pull-steps) ranged from 0–31 (7.67±12.29) and solution times ranged from 9–330 s (83.1±128.39).

Across the 10 standard string-pulling trials the four crows' average pull-step ratio was 90.2±2.42% (including all interactions with the string) and 96.45±1.86% after the first successful pull-step. Therefore, from the first trial crows were chaining together pull and step behaviours into coherent sequences. The four crows only made a total of three errors: (1) Yellow made an uncoordinated third step on the second trial and dropped the string, (2) Yellow also made an uncoordinated second step on the eighth trial and again dropped the string, and (3) Goo made an uncoordinated step on the eighth trial when he failed to pull the string up far enough to step on it.

Crows used two string-pulling techniques: side-stepping and double-stepping. In side-stepping, they moved in one direction along the perch and used the same foot to step on the string. Therefore, they progressively moved further from where the string was tied to the perch. When double-stepping, crows remained stationery and alternately used the right and left foot to step on the string. Owl significantly preferred side-stepping to double-stepping (80% of attempted steps, Binomial choice *p* = 0.002). The other three crows had no preferred stepping technique.

### (b) Experienced Crows and Visually-Restricted String Pulling

Crows in the experienced group took between 1–6 trials (3.0±1.08) to solve the visually-restricted task and obtained the meat between 2–9 times (5.5±1.55) in their 10 trials. Compared to standard string pulling, after the first pull-step the experienced group made 10 times as many errors (One-tailed Wilcoxon signed-ranks test: Z = 10, *p* = 0.0625) and their mean pull-step ratio dropped from 96.5±1.86% to 55.7±10.1% (One-tailed Wilcoxon signed-ranks test: Z = −10, *p* = 0.0625).

The crows made two common errors when they were less than a body length from the hole and could potentially look down at the meat between pull-steps: they performed the first pull-step then stopped (32.4% of total errors) (see Movie S2), and pushed the string against the perch rather than stepping on it (21.6% of errors). They also made two common errors when they were more than a body length from the hole and unable to look down at the meat: making no attempt to step on the string while side-stepping (24.3% of errors), and taking their foot off the string after a pull-step to go and look down the hole (10.8% of errors).

More errors occurred in a pull-step action when a crow did not look down the hole beforehand (errors in 63±1.73% of attempted pull-steps) compared to when they did look down the hole (errors in 9.3±4.62% of attempted pull-steps) (One-tailed Wilcoxon signed-ranks test: Z = −10, *p* = 0.0625). There was a difference in error rates between the two stepping techniques (One-tailed Wilcoxon signed-ranks test: Z = −10, *p* = 0.0625). Only 4.5±fo4.53% of the total attempts at double-stepping failed compared to 57.6±1.46% of side-stepping attempts. Two crows had a significant bias for double-stepping: Goo (90.9%, Binomial choice, *p* = 0.003) and Owl (84.1%, Binomial choice, *p* = 0.026). Yellow and Zola had no preferred stepping technique. Interestingly, Owl switched from a bias for side-stepping in standard string pulling to one for double-stepping in the visually-restricted task.

### (c) Naïve Crows and Visually-Restricted String Pulling

#### (i) Naïve group

One naïve crow, Angel, solved the visually-restricted task in five trials, first doing so on trial 5. Her string-pulling competence developed gradually. In the first two trials Angel only pulled at the string. In her third trial after 17 pulls, she made a pull-step from directly above the hole and then made an unsuccessful side-step. In trial 5 after 40 pulls, Angel obtained the meat after two pull-steps directly above the hole. During this behaviour she also tried to push the string against the perch for the first time. Angel failed in trial 6, pushing the string against the perch six times before attempting to step on it. She was unsuccessful at coordinating pull-steps and again pushed the string against the perch before giving up. Angel successfully obtained the meat in trials 7–10. However, she continued to make a relatively high number of errors by pushing the string against the perch and having problems coordinating pull-step actions.

The other three crows never solved the problem. On his first trial, Boxer made a pull-step directly above the hole but then pushed the string against the perch. In subsequent trials he only ever pulled and then pushed the string against the perch (*n* = 10 times). Robin completed two pull-steps in his final trial, with both steps occurring directly above the hole. However, after the second pull-step he pulled without stepping then left the apparatus. Español pulled at the string 188 times but never stepped on it. Both Robin and Español never pushed the string against the perch.

#### (ii) Mirror group

Two of the four crows solved the visually-restricted task when it was possible to obtain visual feedback via a mirror: Slevin in trial 3 and Ronia in trial 5. Both crows solved the task in six of their 10 trials. Ronia interacted with the string and then looked at the mirror in her first two trials. In the remaining trials she never obviously looked in the mirror during string pulling. However, in all six successful trials she either faced the mirror or had her body sideways-on to it; she was unsuccessful in the only trial with her back towards the mirror. Slevin interacted with the string then obviously looked at the mirror in his first five trials. In his first trial he appeared to see the meat move in the mirror after he pulled the string, and turned to face the mirror while still holding the string. When turning he slid his bill along the string. This bill-sliding became the basis for a novel string-pulling technique which allowed him to pull the meat up with only one pull-step. Slevin did not obviously look in the mirror during his last five successful string-pulling trials. Unlike Ronia, two of Slevin's six successful trials were carried out with his back to the mirror.

Tiga and Egg never solved the task. Tiga appeared distracted by the mirror refection after pulling at the string. She looked at the mirror after interacting with the string in nine of her 10 trials. Although Tiga did not exhibit any obvious startle reactions, on six occasions she left the apparatus after looking in the mirror. Tiga performed one pull-step on her final trial, when directly above the hole, but then left the apparatus. Egg exhibited little or no reaction to the mirror. He completed one pull-step in trial 5 and two in trial 6; the successful pull-steps in trial 6 were interspersed with failed pull-steps, which prevented Egg obtaining the meat.

### (d) Comparison of Visually-Restricted Performances

**Figure 3:**
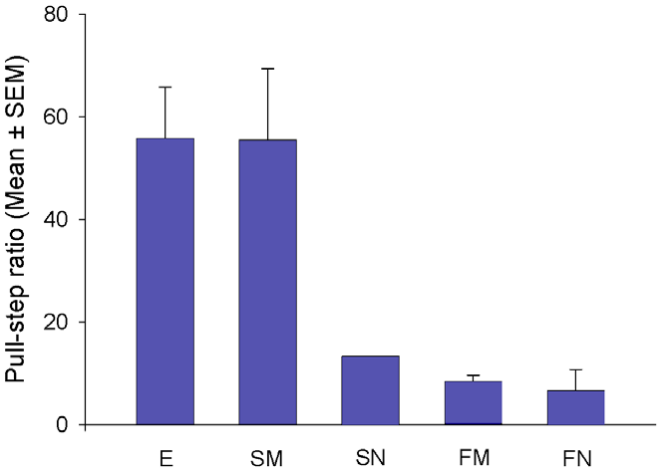
String-pulling efficiency across the three groups with the visually-restricted apparatus. E: experienced group; SM: successful mirror crows; SN: successful naïve crows; FM: failed mirror crows; FN: failed naïve crows.

**Figure 4:**
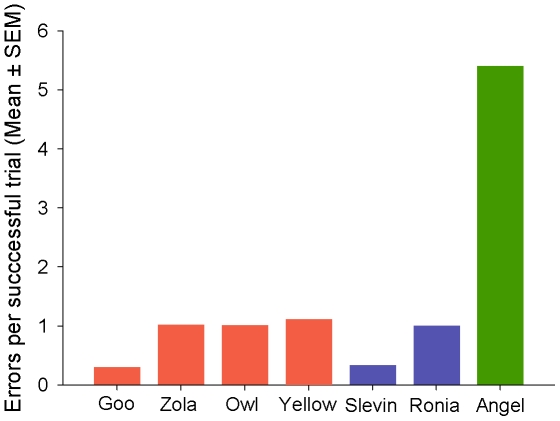
Error rates in successful trials with the visually-restricted task. Red bars: experienced group; Blue bars: mirror group; Green bar: naïve group.

**Figure 5:**
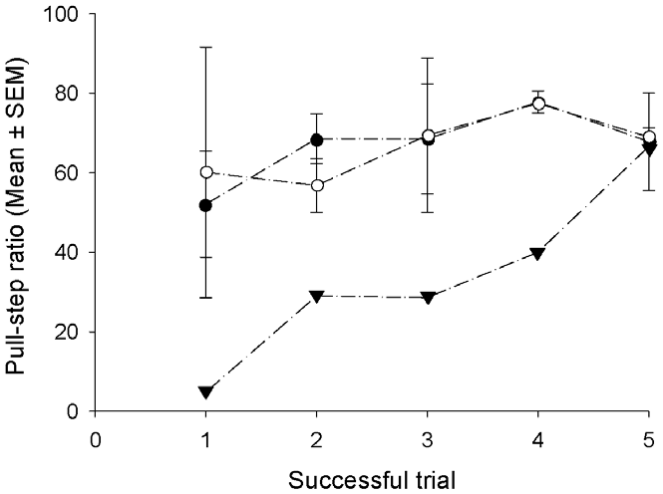
Pull-step ratios in the first five successful trials for the experienced, naïve and mirror groups with the visually-restricted task. White circle: experienced group; Dark circle: mirror group; Triangle: naïve group.

Pull-step ratios were significantly different between the naïve and the experienced groups (Mann-Whitney *U*-test: *U* = 16, *p* = 0.0286). However, they were not different between the mirror group and the experienced group (Mann-Whitney *U*-test: *U* = 10, *p* = 0.6857). These group differences appear to be due to the manner in which the successful crows from the mirror and naïve groups solved the problem. The two successful mirror crows had very similar pull-step ratios and error rates to the experienced group (Figures 3 and 4). In contrast, the one successful naïve crow had a pull-step ratio three times lower than that of the successful crows in the other two groups and made five times as many errors during successful trials (Figures 3 and 4). The low number of successful individuals in the naïve (*n* = 1) and mirror (*n* = 2) groups precludes between-group statistical comparisons of pull-step ratios and errors for these three successful crows. However, changes in pull-step ratios across successful trials could be analysed. This analysis showed that trial number did not predict success for the mirror and the experienced groups (Linear regression: *R^2^* = 0.001, *F*_1,8_ = <0.001, *p* = 0.99 and *R^2^* = 0.11, *F*_1,15_ = 1.83, *p* = 0.20, respectively), but it did for the successful naïve crow Angel (*R^2^* = 0.903, *F*_1,3_ = 28.068, *p* = 0.013) (Figure 5). Angel's pull-step ratio increased across successful trials (Regression coefficient  = 13.46: *t*_1,3_ = 5.298, *p* = 0.013). The gradual increase within these trials and the high number of errors suggests that Angel solved the problem by trial and error learning.

### (e) String Discrimination

All 12 crows significantly preferred to pull the string with the meat attached rather than the string with a rock attached. In 90.8% of the 240 trials they chose the former string (Binomial choice, *p* = 0.0002). Only Tiga, the juvenile, chose the rock on the first trial. The crows performed at similar levels with the slanted string, although three of them chose the rock on the first trial. The 12 crows chose the slanted string with food in 89.1% of the 240 trials (Binomial choice, *p* = 0.0002). The eight crows performed well on the overload test. Six of them chose the small reward on the first trial and across all eight crows this reward was chosen in 93% of the 160 trials (Binomial choice, *p* = 0.0002).

Performances dramatically declined in all three crossed-string conditions. Six of the 12 crows chose the string with food on the first trial; one crow in the same-colour condition, three in the different-colour condition and two in the patterned-colour condition. In the same-colour condition, the four crows chose the correct string in 43.8% of the 80 trials (Binomial choice, *p* = 0.317). Crows performed slightly better in the different-colour condition, choosing the correct string in 55% of the 80 trials (Binomial choice, *p* = 0.435). In the patterned-colour condition, the four crows had a significant bias for the string above the meat rather than the one connected to it (correct string chosen in 32.5% of the 80 trials; Binomial choice, *p* = 0.002). The performance of the crossed-string groups was significantly better in the last 5 trials (58.3±2.32% correct) than in the first 5 trials (38.3±3.47% correct) (Paired t-test t = −2.345, *p* = 0.039), which suggested that some learning occurred.

## Discussion

Three of the four New Caledonian crows initially tested on the standard string-pulling problem solved the task on the first trial. Two of these three crows performed a sequence of pull-step actions without making any other string-orientated behaviour beforehand. Such spontaneity has not been shown by ravens naïve to string pulling [7], [8], but has been seen in the performances of keas [10]. The ‘insight’ hypothesis assumes that the string-pulling problem is mentally solved by an animal either before interaction or after the first pull-step has been completed [8]. Nevertheless, even after the first pull-step on the visually-restricted apparatus, the crows experienced at standard string pulling showed a drop in performance and an increase in error rates. Similarly, the three naïve crows that completed at least one pull-step with this apparatus did not then spontaneously chain these behaviours together to solve the problem. These results are not consistent with the hypothesis that the crows built a mental scenario, either during their successful solution of the standard string-pulling problem or after the first pull-step on the visually-restricted apparatus. However, due to the low sample size further work investigating the effect of interrupting visual feedback is required.

The crows also did not appear to have had any insight into the relation between the string and the reward [6], [12], [13]. Although the crows were able to solve a number of low-level string discrimination tasks, their performance dropped on the more complex crossed-string tasks. The crows were not able to consistently choose the string baited with meat, even when the two strings differed in colour or pattern. This suggests that they did not have an understanding of ‘connectivity’, the causal relation between the string and the meat. This failure may be due to the crows lacking sufficient exposure to string pulling. They were given 60 string-pulling trials before the crossed-string problems and the string had been presented in a variety of arrangements in these trials. In a recent study, New Caledonian crows only became sensitive to a causal relation after an average of over 100 trials with the problem [20], [21].

The behaviour of the experienced group of crows with the visually-restricted apparatus supports the hypothesis that operant conditioning mediated by perceptual-motor feedback is important for string pulling. Even though these crows had already completed 10 trials of standard string pulling, they made more errors and chained pull-step behaviours together with less efficiency when faced with the visually-restricted problem. They also made more errors when they did not look down the hole before a pull-step action. One crow even changed its string-pulling technique from a side-stepping one to a double-stepping one, which allowed it to continually look down the hole during string pulling.

The evidence from the naïve and mirror groups for the use of visual feedback in string pulling is weaker than that from the experienced group. Although three of the four naïve crows were unable to solve the problem, one naïve crow was successful. Also, only two of the mirror crows were successful when visual feedback was potentially available. The low numbers of successful crows in these groups constrained the statistical analyses that we could carry out. The two successful mirror crows had similar error rates and proficiency to the four crows in the experienced group, despite being naïve to string pulling. In contrast, the successful naïve crow Angel made many more errors and had a much lower pull-step ratio. The gradual increase in proficiency over time seen in Angels' performance is suggestive of trial and error learning. One reason for the weak performance of the two unsuccessful crows in the mirror group might be because the mirror distracted them. One of these two crows, Tiga, left the apparatus in six trials after looking into the mirror and did not return.

There are two ways in which the visually-restricted apparatus could have affected perceptual-motor feedback. First, the apparatus could disrupt visual feedback because the meat moved out of sight during string pulling. Experienced crows made fewer errors in the visually-restricted task when they looked down the hole before attempting a pull-step. They made more errors when they used a side-stepping technique, which took them progressively further from the hole. However, neither pulling nor stepping on the string was dependent on the meat being in view, eliminating the use of a simple ‘out of sight, out of mind’ perceptual-motor feedback mechanism.

Second, the quality of visual feedback could have been reduced even when crows were looking through the hole at the meat. Humans find it difficult to judge whether an object is moving closer when the angle of approach is zero, that is, when the object is coming straight towards the observer [22]. Crows viewing the meat through the hole had only this head-on perspective of the effect of their actions on the meat. Therefore, it may have been difficult for them to judge which string-directed behaviour moved the meat and returned it to its original position (a pull-drop), and which behaviour moved the meat and kept it slightly closer than before (a pull-step). Determining if the meat had moved closer would have been most difficult after the first pull-step as crows would need to judge from a relatively long distance whether the meat had moved a few centimetres towards them or not. A common error made by the experienced crows was to complete a single pull-step and then stop string pulling. The successful mirror crows never made such an error. This difference supports the hypothesis that side-on visual feedback via the mirror allowed crows to detect if the meat moved closer after the first pull-step and so reinforced these actions.

Our findings here raise the possibility that string pulling is based on operant conditioning mediated by a perceptual-motor feedback cycle rather than on ‘insight’ or causal knowledge of string ‘connectivity’. However, as only two crows were successful in the mirror group the results are not conclusive and further testing with a larger sample size is required. One issue must be accounted for if perceptual-motor feedback is to be considered a plausible explanation for spontaneous string pulling. All the bird species tested so far would have been able to learn via operant conditioning, yet large inter-species differences have been found. Adult European greenfinches and canaries are unable to solve the standard string-pulling problem [2]. Juveniles of these species can only solve the string-pulling problem if the baited string is gradually lengthened across trials. Similarly, only 6% of goldfinches and 35% of siskins solved the standard string-pulling task within the first hour of exposure to it [4]. Both goldfinches and siskins use their feet in the wild for holding buds, seeds and grass stems [23]. Therefore, spontaneous string pulling requires more than this behavioural prerequisite. To our knowledge, spontaneous string pulling by naïve birds has been found only in psittacids and corvids. These two families have enlarged forebrains in comparison to other birds [24], particularly in the nidopallium and mesopallium regions [25], [26]. A possible explanation for the inter-species differences, if string pulling is based on operant conditioning, is that bird species with larger associative brain areas are able to integrate information between perceptual and motor pathways quicker than species with smaller associative brain areas. That is, they can quickly identify novel actions that have a positive effect when in the process of creating novel sequences of behaviour. This is compatible with an ‘embodied cognition’ perspective (see [27] for a full definition). Embodied cognition involves an animal developing complex behaviour through understanding the consequences of its own actions, without using off-line processes such as insight and planning [27], [28]. This cognition is similar to that involved in time-pressured human spatial decision making such as steering a car or playing a computer game like Tetris [29]. The perceptual-feedback hypothesis, therefore, could potentially account for the interspecies variation found so far. This hypothesis makes empirical predictions concerning the role of perceptual feedback in string pulling, the ability of moving food to act as an internal psychological reinforcer and the link between information integration and behavioural flexibility. Testing these predictions against those of the insight hypothesis will be necessary to shed light on the actual cognitive mechanisms underpinning spontaneous string pulling.

## Supporting Information

Movie S1 First trial of Zola solving the standard string-pulling problem.
(0.91 MB MOV)

Movie S2 Second trial of Zola performing a single pull-step on the visually-restricted problem.
(2.50 MB MOV)
